# GPX4 suppresses ferroptosis to promote malignant progression of endometrial carcinoma via transcriptional activation by ELK1

**DOI:** 10.1186/s12885-022-09986-3

**Published:** 2022-08-12

**Authors:** Sitian Wei, Zhicheng Yu, Rui Shi, Lanfen An, Qi Zhang, Qian Zhang, Tangansu Zhang, Jun Zhang, Hongbo Wang

**Affiliations:** Department of Gynecology and Obstetrics, Union Hospital, Tongji Medical College, Huazhong University of Science and Technology, Wuhan, Hubei People’s Republic of China

**Keywords:** Endometrial carcinoma, GPX4, ELK1, Ferroptosis, Transcriptional activation

## Abstract

**Background:**

Glutathione Peroxidase 4 (GPX4) is a key protein that inhibits ferroptosis. However, its biological regulation and mechanism in endometrial cancer (EC) have not been reported in detail.

**Methods:**

The expression of GPX4 in EC tissues was determined by TCGA databases, qRT-PCR, Western blot, and immunohistochemistry (IHC). The effects of GPX4 on EC cell proliferation, migration, apoptosis, and tumorigenesis were studied in vivo and in vitro. In addition, ETS Transcription Factor ELK1 (ELK1) was identified by bioinformatics methods, dual-luciferase reporter assay, and chromatin immunoprecipitation (ChIP). Pearson correlation analysis was used to evaluate the association between ELK1 and GPX4 expression.

**Results:**

The expression of GPX4 was significantly up-regulated in EC tissues and cell lines. Silencing GPX4 significantly inhibited the proliferation, migration ability, induced apoptosis, and arrested the cell cycle of Ishikawa and KLE cells. Knockdown of GPX4 accumulated intracellular ferrous iron and ROS, disrupted MMP, and increased MDA levels. The xenograft tumor model also showed that GPX4 knockdown markedly reduced tumor growth in mice. Mechanically, ELK1 could bind to the promoter of GPX4 to promote its transcription. In addition, the expression of ELK1 in EC was positively correlated with GPX4. Rescue experiments confirmed that GPX4 knockdown could reverse the strengthens of cell proliferation and migration ability and the lower level of Fe^2+^ and MDA caused by upregulating ELK1.

**Conclusion:**

The results of the present study suggest that ELK1 / GPX4 axis plays an important role in the progress of EC by promoting the malignant biological behavior and inducing ferroptosis of EC cells, which provides evidence for investigating the potential therapeutic strategies of endometrial cancer.

**Supplementary Information:**

The online version contains supplementary material available at 10.1186/s12885-022-09986-3.

## Introduction

Endometrial carcinoma (EC) is one of the common malignant tumors in gynecology, threatening women's health and life, and increasing the disease risk and social medical burden [1]. According to the latest global cancer burden data in 2020 released by the international agency for research on cancer (IARC) of the World Health Organization, endometrial carcinoma is the sixth most common female cancer, with 417,000 new cases and 97,000 deaths in 2020 [2]. Most patients can get an early diagnosis and have a good prognosis [3]. However, despite increasing treatment options currently available [4, 5], outcomes for advanced, poorly differentiated, or special types of EC, such as high-grade EC and EC with papillary serous or clear cell histology, are still dismal [6, 7]. More than that, early or well-differentiated endometrioid tumors also have an unexpected recurrence and poor prognosis[8]. Therefore, exploring the pathogenesis and effective therapeutic targets of EC is urgently required to improve the prognosis for patients with EC.

Ferroptosis, a newly discovered form of programmed cell death, depends on the accumulation of excessive iron in cells and causes the increase of toxic lipid peroxide ROS [9, 10]. The main mechanism of ferroptosis is that under the action of divalent iron or ester oxygenase, it catalyzes the unsaturated fatty acids highly expressed on the cell membrane to produce lipid peroxidation and induce cell death [11, 12]; In addition, the expression of the antioxidant system (glutathione GSH and glutathione peroxidase 4 GPX4) decreased [13, 14]. Glutathione peroxidase 4 (GPX4) belongs to the glutathione peroxidase family, whose members catalyze the reduction of hydrogen peroxide, organic hydrogen peroxide, and lipid hydrogen peroxide, so GPX family to protect cells from oxidative damage [15]. At present, several members such as GPX1-GPX8 have been found in mammals [16, 17]. However, only GPX4 showed the ability to scavenge membrane lipid hydrogen peroxide products, which is also related to its unique amino acid sequence and spatial structure [18, 19]. Some studies have shown that the expression level of GPX4 in tumor tissues is significantly higher than that in normal tissues [20], such as renal clear cell carcinoma [21], lung adenocarcinoma [22], prostate cancer [23], thyroid cancer [24], and gastric cancer [25]. It is speculated that GPX4 may be an oncogene. However, the potential carcinogenic function of GPX4 on EC and the potential mechanism of the association between GPX4 and EC have not been reported.

To solve this problem, we first evaluated the expression of GPX4 in EC through public databases and experiments such as immunohistochemistry (IHC). To further study the function of GPX4 in EC, a series of experiments in vitro were carried out to confirm the carcinogenic effect of GPX4 in EC. It was found that inhibiting GPX4 can significantly inhibit cell proliferation, migration, promote apoptosis and block the cell cycle of EC cells. Besides, by detecting a series of indicators reflecting ferroptosis, we found that knocking down GPX4 could induce ferroptosis in EC cells. Moreover, the studies of mice models in vivo showed that knockdown of GPX4 significantly reduced the volume and weight of tumors and enhanced ferroptosis activity. In addition, we found that GPX4 can be used as a new transcriptional target of ELK1, and the tumor progression caused by the overexpression of ELK1 can be attenuated by downregulating the expression of GPX4. In conclusion, our results provide new insights into the progress of GPX4 in EC and provide a new theoretical basis for the prevention and treatment of patients with endometrial cancer.

## Materials and methods

### Cell culture

The human endometrial carcinoma cell line Ishikawa and KLE were grown in DMEM-F12 complete medium (HyClone, China) supplemented with 10% fetal bovine serum (Gibco, USA) and 1% antibiotics (penicillin and streptomycin) (Boster, China) at a final concentration of 100 µg/ml. All cells were grown at 37 ℃ in a humidified atmosphere of a 5% CO_2_ incubator. All the cells were free of mycoplasma contamination.

### Transfection

Lentivirus vectors containing GPX4-shRNA and shNC were purchased from GenePharma (Shanghai, China). Two shRNA sequences of GPX4 were: shGPX4-1: sense 5'-CACCGTGGATGAAGATCCAACCCAATTCAAGAGATTGGGTTGGATCTTCATCCACTTTTTTG-3'; antisense 5'-GATCCAAAAAAGTGGATGAAGATCCAACCCAATCTCTTGAATTGGGTTGGATCTTCATCCAC-3'; shGPX4-2: sense 5'-CACCGCACATGGTTAACCTGGACAATTCAAGAGATTGTCCAGGTTAACCATGTGCTTTTTTG-3'; antisense 5'-GATCCAAAAAAGCACATGGTTAACCTGGACAATCTCTTGAATTGTCCAGGTTAACCATGTGC-3'. Fourty-eight hours after transfection, qRT-PCR and WB were used to examine the GPX4-shRNA interference effect. Stable cells transfected with GPX4-shRNA were obtained after 14 days of puromycin screening (2 μg/ml).

### Clinical specimens

A total of six paired EC and adjacent tissues of patients who underwent surgery or biopsy in the Department of Gynecology, Union Hospital Affiliated to Tongji Medical College, Huazhong University of Science and Technology (Wuhan, China) from September 2019 to March 2021 were collected for qRT-PCR, IHC and western blotting. All patients had complete clinical data and did not receive immunotherapy, chemotherapy, or radiotherapy. This study was approved by the Ethics Committee of Tongji Medical College, Huazhong University of Science and Technology (No. 2022-S017). All patients have signed the written informed consent forms before surgical resection.

### Immunohistochemistry (IHC) staining and scoring

Samples were embedded in paraffin and sliced into sections at a thickness of 4 μm. The sections were then incubated with GPX4 antibody (Proteintech, China) which was diluted at 1: 3000 at 4 ℃ overnights. The following day, the stained sections were incubated with secondary antibody at room temperature for 30 min in darkness and visualized with DAB solution. Finally, the nucleus was counterstained by haematoxylin. A Motic microscope (Motic, China) was used to visualize and photograph the sections. The IHC staining scores were evaluated by two independent observers blinded to the corresponding patients based on the staining intensity (SI) and the percentage of immunoreactive cells (PR) [26]. The SI score was calculated from 0 to 3: 0 = no staining; 1 = weak staining; 2 = moderate staining; and 3 = strong staining. The PR was scored from 1 to 4: 1 = 0–25%; 2 = 26–50%; 3 = 51–75%; and 4 = 75–100%. The PR and the SI were multiplied to produce a weighted score for each patient. A score of 8–12 was defined as a high expression level and a score of 0–7 was defined as low expression.

### Total RNA isolation and quantitative real-time polymerase chain reaction (qRT-PCR)

The adherent cells were harvested with the RNAiso reagent (Takara, Japan). The extraction of RNA was performed according to the manufacturer's manual. The cDNA was obtained from RNA using the reverse transcription kit (Vazyme, China). The cDNA was used as templates, and the SYBR Green Fast qPCR Mix (Abclonal, China) was used for real-time quantitative PCR. The fold changes of RNA transcripts were calculated by the 2 − ΔΔCt method and glyceraldehyde 3-phosphate dehydrogenase (GAPDH) was utilized as the internal control. The primer sequences used were shown as follows: GPX4-F: 5’-GAGGCAAGACCGAAGTAAACTAC-3’; GPX4-R: 5’-CCGAACTGGTTACACGGGAA-3’; GAPDH-F: 5’-GAGTCAACGGATTTGGTCGT-3’; GAPDH-R: 5’-GACAAGCTTCCCGTTCTCAG-3’.

### Western blotting

Fresh tissues or adherent cells were first washed twice with PBS and then lysed on ice with RIPA lysis buffer (Servicebio, China) containing Cocktail and PMSF for 1 h. Cell lysates were centrifuged and quantified using BCA Protein Assay kits (Biosharp, China). A total of 30 µg of protein was loaded for electrophoretic separation on sodium dodecyl sulfate (SDS)-polyacrylamide gels and transferred onto polyvinylidene difluoride (PVDF) membranes. After incubation with 5% skimmed milk at room temperature for 1 h and washing, the membranes were further incubated with primary antibody at 4 ℃ overnight. Membranes were blotted with the following antibodies: anti-GPX4 (1: 1000; Proteintech, China), anti-ELK1 (1: 1000; Proteintech, China), and anti-GAPDH (1: 10,000; Abclonal, China). Horseradish peroxidase-conjugated secondary antibody was added and incubated at room temperature for 60 min, followed by capture with enhanced chemiluminescence (ECL) (Biosharp, China).

### Cell proliferation assay

Cell Counting Kit-8 (CCK8) assay (Targetmol, China) was used to detect cell viability. A total of 100 μl cell suspension (3 × 10^3^ cells) was first plated in a 96-well plate, and then 10 μl of CCK8 solution was added to each well at 24, 48, 72, 96, 120 h. After another 2 h of incubation, the absorbance was measured at 450 nm by using a microplate reader (Thermo Fisher Scientific, USA). Clone formation assay was used to detect the number of colonies. Cells were seeded into well plates (300 cells per well) and cultured for 14 days. The above-mentioned cells were then fixed for 30 min with 2 ml of methanol and then stained cells with crystal violet solution for about 30 min.

### Cell migration assays

Transwell chambers of 8 μm pore size (Corning, USA) were used to assess migration. 100 µl cell suspension without serum was used in the upper chamber with Ishikawa and KLE (1.0 × 10^5^/well). The lower chamber contained a chemo-attractant (medium with 20% FBS). After 24-h incubation at 37 ℃, the cells were then fixed with 4% paraformaldehyde (Servicebio, China) for 30 min and stained with 0.1% violet crystal (Servicebio, China) for 10 min at room temperature. After washing with PBS solution, cells that passed the membrane were counted and imaged in 3 different randomly selected views under 100 × magnification using CX23 Olympus light microscopy (Olympus, Japan).

### Flow cytometry (FCM) analysis

Use the Annexin V-FITC Apoptosis Detection Kit (Beyotime, China) to evaluate cell apoptosis. Use PI/RNase Staining Buffer (BD Biosciences, USA) to analyze the cell cycle. The flow cytometer used was ID7000 Spectral Cell Analyzer (Sony, Japan). The data were analyzed by using FlowJo.

### Lipid reactive oxygen species (ROS) assay

Cells were collected and then centrifuged. After that, DCFH-DA (Beyotime, China) was prepared to pretreat the cells at 37 ℃ for 20 min. Following incubation, the cells were collected and washed twice in a free-serum medium. Subsequently, the cells were resuspended in PBS and were analyzed by flow cytometry to detect the levels of ROS within the cells.

### Fe2 + and malondialdehyde (MDA) assay

Firstly utilized a BCA protein assay kit (Biosharp, China) to detect the protein concentrations in cell supernatants. After that, Fe^2+^, as well as the MDA levels, were both estimated by the iron assay kit (NJJCBIO, China) and the MDA detection kit (Beyotime, China), respectively.

### Evaluation of mitochondrial membrane potential (MMP)

The MMP was measured by a mitochondrial membrane potential assay kit (Beyotime, China). JC-1 monomers (green) can form aggregates (red fluorescence) in the mitochondria with high △Ψm, which cannot form aggregates in the mitochondria with low △Ψm. Results were detected by using a confocal laser microscope (Olympus, Japan).

### Dual-luciferase reporter assay

Ishikawa cells were seeded in 6-well plates at a 70% concentration and transfected with relevant plasmid and the luciferase vector. After 48 h, cells were lysed and the luciferase activities were measured using the dual-luciferase reporter assay kit (Beyotime, China) according to the manufacturer’s protocol. The luminescence intensities of firefly and Renilla luciferases were recorded by a microplate reader. For data analysis, the luciferase activity was normalized by comparing it with Renilla luciferase activity.

### Chromatin immunoprecipitation (ChIP) assay

Chromatin immunoprecipitation was performed using Ishikawa cells, which were treated with 37% formaldehyde (Sigma, USA) at 1% final concentration for 10 min at room temperature to cross-link proteins to DNA. After that, the remaining steps were carried out according to the manufacturer’s instructions for the ChIP assay kit (Beyotime, China). The antibodies used were anti-ELK1 (Proteintech, China) and normal rabbit IgG (Proteintech, China). The enriched DNA was analyzed utilizing the primers by real-time PCR.

### Tumor xenograft assay

Female BALB/c-nu nude mice (age, 4–5 weeks)were purchased from China Charles River and housed in a standard pathogen-free environment laboratory. The mice were randomized into two groups. Ishikawa (1 × 10^6^) were suspended in 100 μl serum-free DMEM and subsequently injected into the right flanks of the mice. After 25 days, the mice were cervical dislocated under anesthesia, and the weight, length, and width of xenografts were measured. Tumor volume = (length × width^2^)/2. Tumor samples were partially embedded in paraffin for histopathological analysis. Animal experiments were performed according to the protocols approved by Tongji Medical College's Animal Care and Use Committee (No. 2021-S2783).

### Statistical analysis

GraphPad Prism 8 (GraphPad Software, USA) was used for statistical analysis. The experimental data of every 3 independent replicates were presented as the mean ± standard error of the mean (SEM). The Student's t-test was used for comparisons between two independent sample groups. One-way ANOVA was used for one-way comparisons between multiple groups, while two-way ANOVA tests were used for two-way comparisons between multiple groups. P < 0.05 was considered to indicate a statistically significant difference. * *P* < 0.05, ** *P* < 0.01, *** *P* < 0.001, **** *P* < 0.0001.

## Results

### GPX4 is highly expressed in EC tissues

**Fig. 1:**
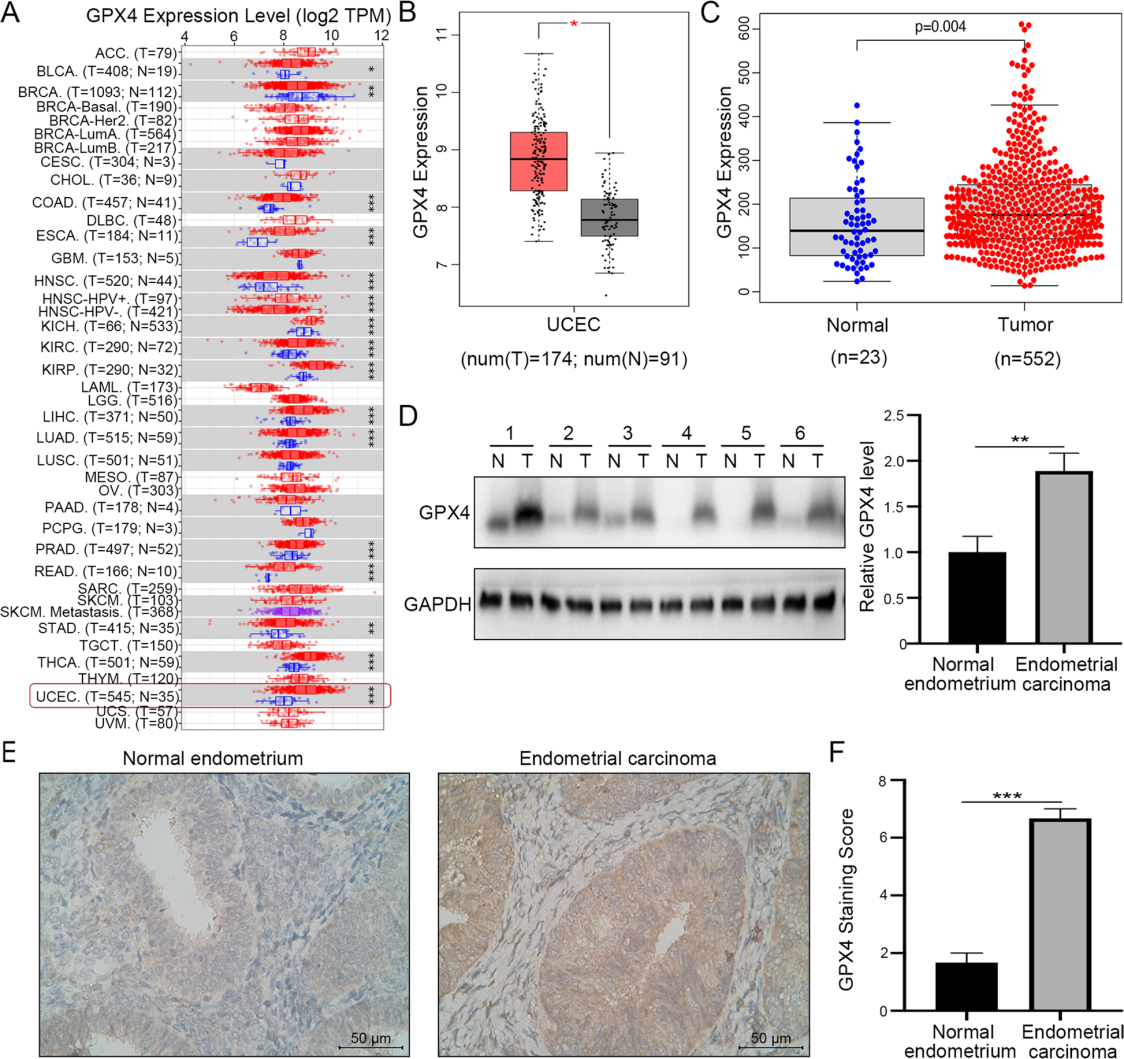
GPX4 expression is elevated in EC. **A-C** The mRNA expression of GPX4 in endometrial tumor tissues and normal endometrial tissues was analyzed based on TIMER, GEPIA, and TCGA databases. **D** GPX4 protein expression in EC tissues and adjacent normal tissues was analyzed by western blot. **E** Representative immunohistochemistry (IHC) images of GPX4 in EC tissues and adjacent normal tissues. Scale bar: 50 μm. **F** The protein expression of GPX4 in tumor and adjacent normal tissues from EC patients was detected by IHC

In this study, we first explored the expression levels of GPX4 in endometrial cancer tumor tissues and normal endometrial tissues in TIMER and GEPIA databases. It was found that the expression of GPX4 in EC tumor tissues was higher than that in normal endometrial tissues (*P* < 0.05) (Fig. 1A, B). We performed data mining and analyzed GPX4 mRNA profiles from the publicly available TCGA databases. The results showed that the expression of GPX4 in EC tumor tissues was significantly elevated than that in normal endometrial tissues (*P* < 0.05, Fig. 1C). Next, six pairs of tumor specimens from EC patients and their corresponding adjacent normal tissues were analyzed by Western blot and immunohistochemical (IHC) staining. Western blot results indicated that the expression level of GPX4 protein in EC tumor tissue was significantly higher than that in normal endometrium (Fig. 1D). We scored the GPX4 expression level based on the GPX4 staining intensity and the percentage of positive tumor cells. The IHC score of GPX4 expression was also significantly increased in EC tumor tissues than the noncancerous counterparts (Fig. 1E, F). Overall, these results demonstrated that GPX4 expression was elevated in tumor tissues of patients with endometrial cancer.

### GPX4 knockdown suppresses proliferation, migration, induces apoptosis, and arrests cell cycle progression of EC cells in vitro

**Fig. 2:**
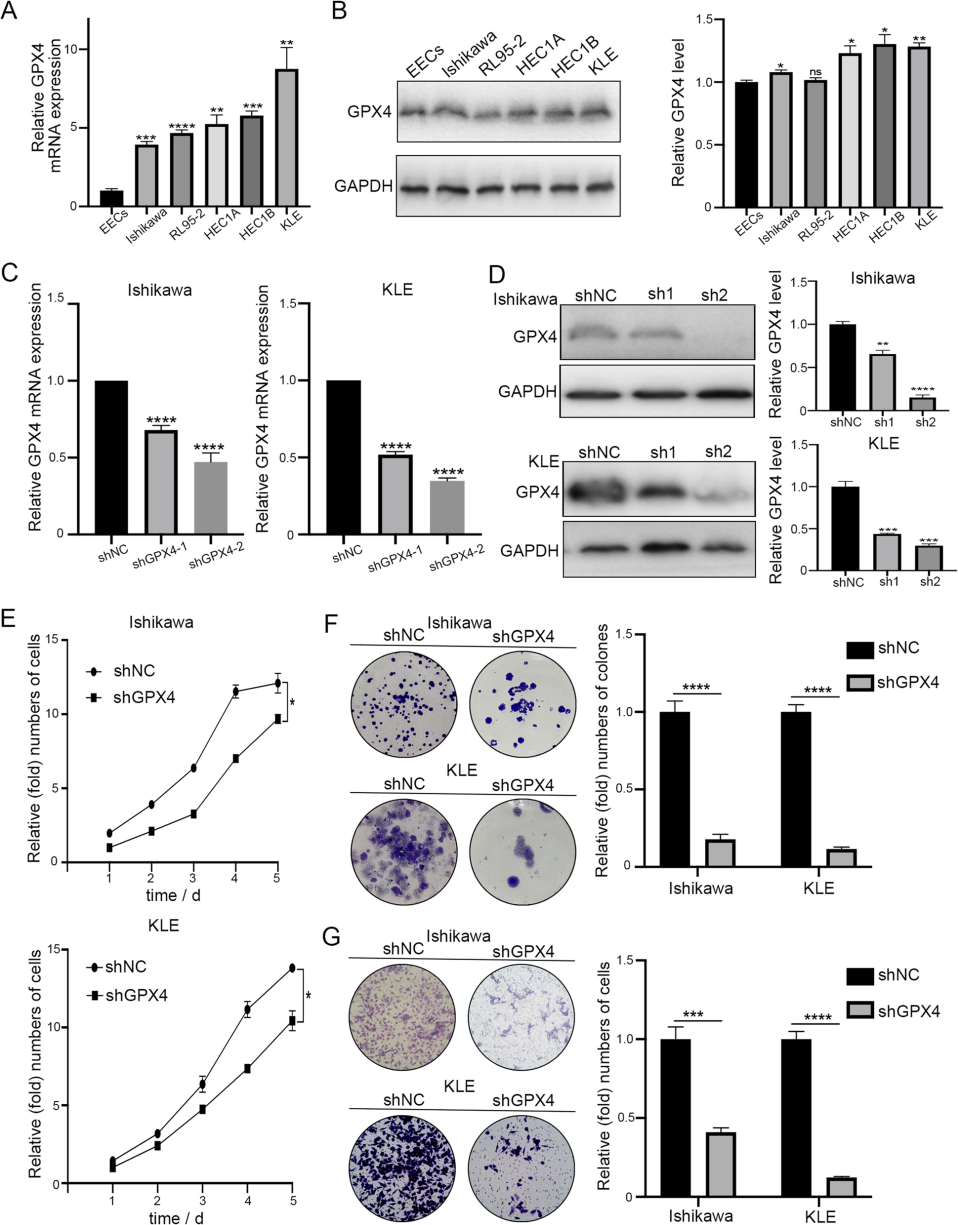
Knockdown of GPX4 suppresses cell proliferation and migration of EC cells in vitro. **A**,** B** GPX4 mRNA and protein expression in human primary endometrial epithelial cells (EECs) and EC cell lines was analyzed by qRT-PCR and western blot, respectively. **C** The knockdown efficiency of shGPX4 was detected by qRT-PCR in Ishikawa and KLE cells. **D** The knockdown efficiency of shGPX4 for following experiments was validated by western blot. **E**,** F** Cell proliferation was detected by CCK8 and colony formation assay following GPX4 knockdown in EC cells. **G** Cell migration ability was determined by transwell assay following GPX4 knockdown in EC cells

**Fig. 3:**
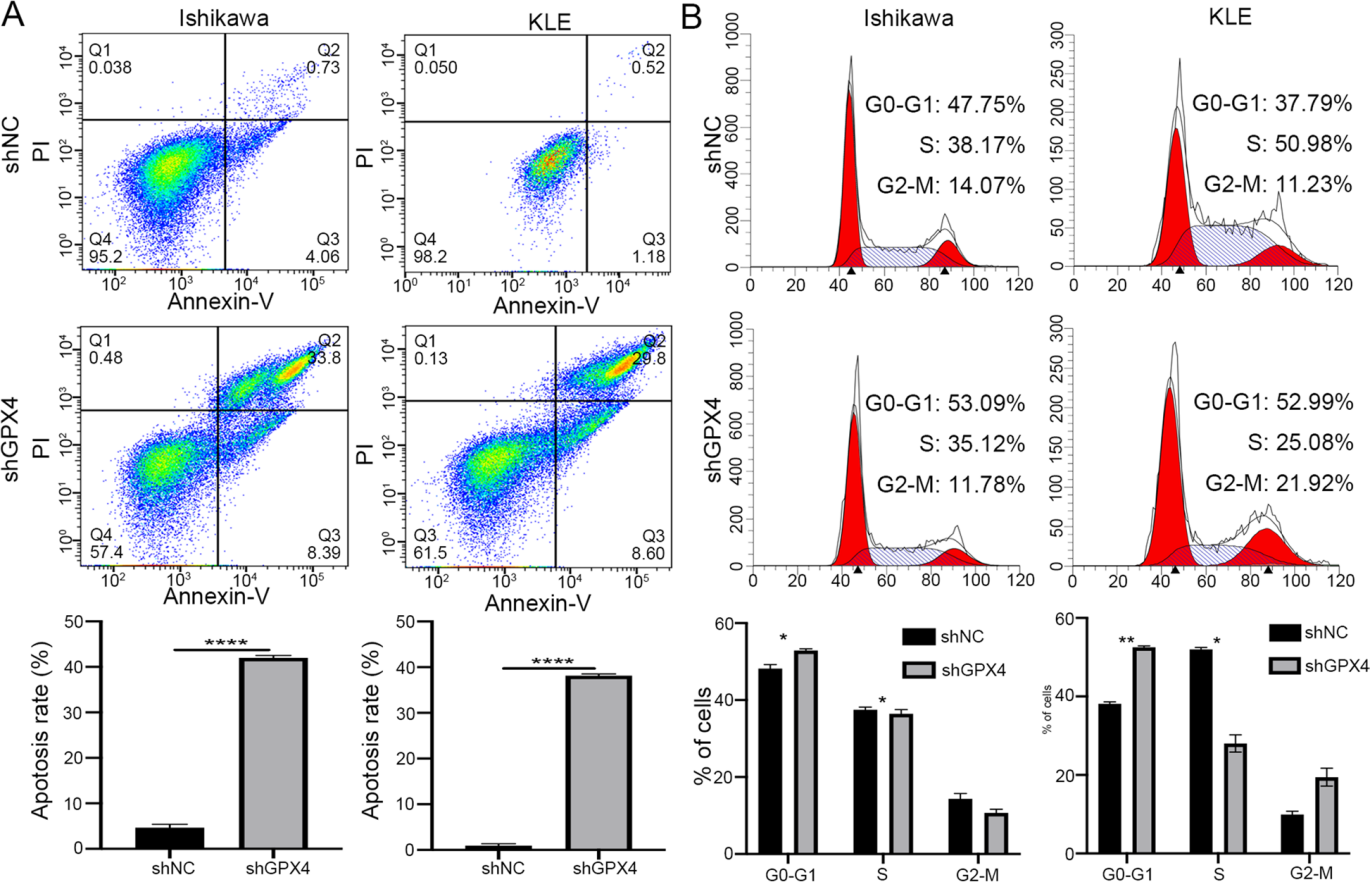
Knockdown of GPX4 induces apoptosis, and arrests cell cycle progression of EC cells in vitro. **A** Cell apoptosis of EC cells following GPX4 knockdown was analyzed by flow cytometer. **B** The cell cycles of shNC and shGPX4 cells were analyzed using flow cytometry

To investigate the functional role of GPX4 in EC cells, we firstly measured GPX4 mRNA and protein levels in human primary endometrial epithelial cells (EECs) and several EC cell lines (Fig. 2A, B). GPX4 was proved to be highly expressed in EC cell lines and we selected Ishikawa and KLE cell lines for further studies. Two shRNAs (shGPX4-1 and shGPX4-2) were designed and synthesized to knock down GPX4 in both cell lines (Fig. 2C). The shRNA (shGPX4-2) that exhibited higher interference efficiency was confirmed by western blotting and selected for the subsequent experiments (Fig. 2D). The expression of GPX4 in Ishikawa and KLE cells was significantly suppressed compared with the shNC group. CCK8 and colony formation assays indicated that GPX4 silencing significantly impeded the viability and cloning ability of both Ishikawa and KLE cells (Fig. 2E, F). The capability of cell migration was also significantly decreased after GPX4 knockdown by transwell assay (Fig. 2G). Moreover, we also observed dramatically increased apoptotic cells in shGPX4 group compared with that in shNC group (Fig. 3A). Meanwhile, we performed FACS for cell cycle analysis on GPX4-knockdown cells and their control cells. Compared with shNC group, the proportion of shGPX4 cells in the G0/G1 phase sharply increased, while the proportion in the S phase abruptly decreased (Fig. 3B). In summary, these findings confirm that loss of GPX4 could suppress proliferation, migration, induce apoptosis, and arrest cell cycle progression from the G0/G1 phase to the S phase in EC cells.

### GPX4 knockdown induces cell ferroptosis in EC

**Fig. 4:**
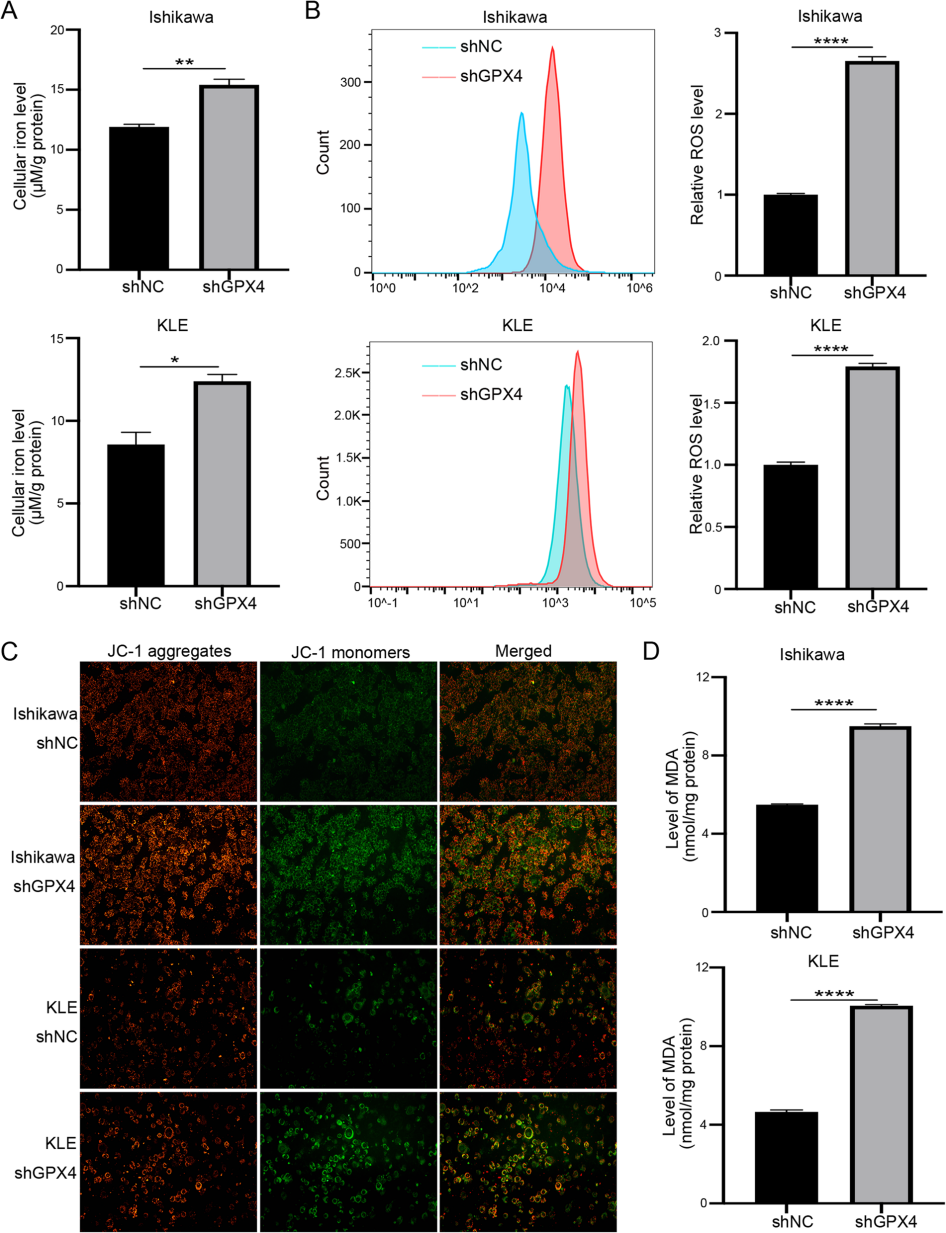
GPX4 knockdown induces ferroptosis in EC. **A** Intracellular ferrous iron levels were determined via the iron assay in Ishikawa and KLE cells silencing or not silencing GPX4. **B** Lipid ROS levels were measured via flow cytometric analysis in Ishikawa and KLE cells silencing or not silencing GPX4. **C** MMP was detected via JC-1 staining in Ishikawa and KLE cells silencing or not silencing GPX4. **D** MDA levels were measured in Ishikawa and KLE cells silencing or not silencing GPX4

Accumulating evidence has shown that cell ferroptosis is mainly caused by elevated intracellular lipid peroxidation disrupting mitochondrial membrane density, and by an accumulation of lethal ROS during iron metabolism. In the present study, the level of intracellular Fe^2+^ notably increased in shGPX4 group of both Ishikawa and KLE cells compared with shNC group (Fig. 4A). Similarly, lipid ROS levels in the two EC shGPX4 cell lines increased (Fig. 4B). The JC-1 staining indicated that shGPX4 group significantly increased monomeric JC-1, which showed mitochondrial oxidative damage (Fig. 4C). Moreover, both Ishikawa and KLE cells knocked down with GPX4 indicated a rise in MDA levels, which is one of the most vital end-products of lipid peroxidation (Fig. 4D). Collectively, these results indicated that knocking down GPX4 significantly increased ferroptosis in EC cells.

### GPX4 promotes EC development, and GPX4 knockdown enhances ferroptosis activity in vivo

**Fig. 5:**
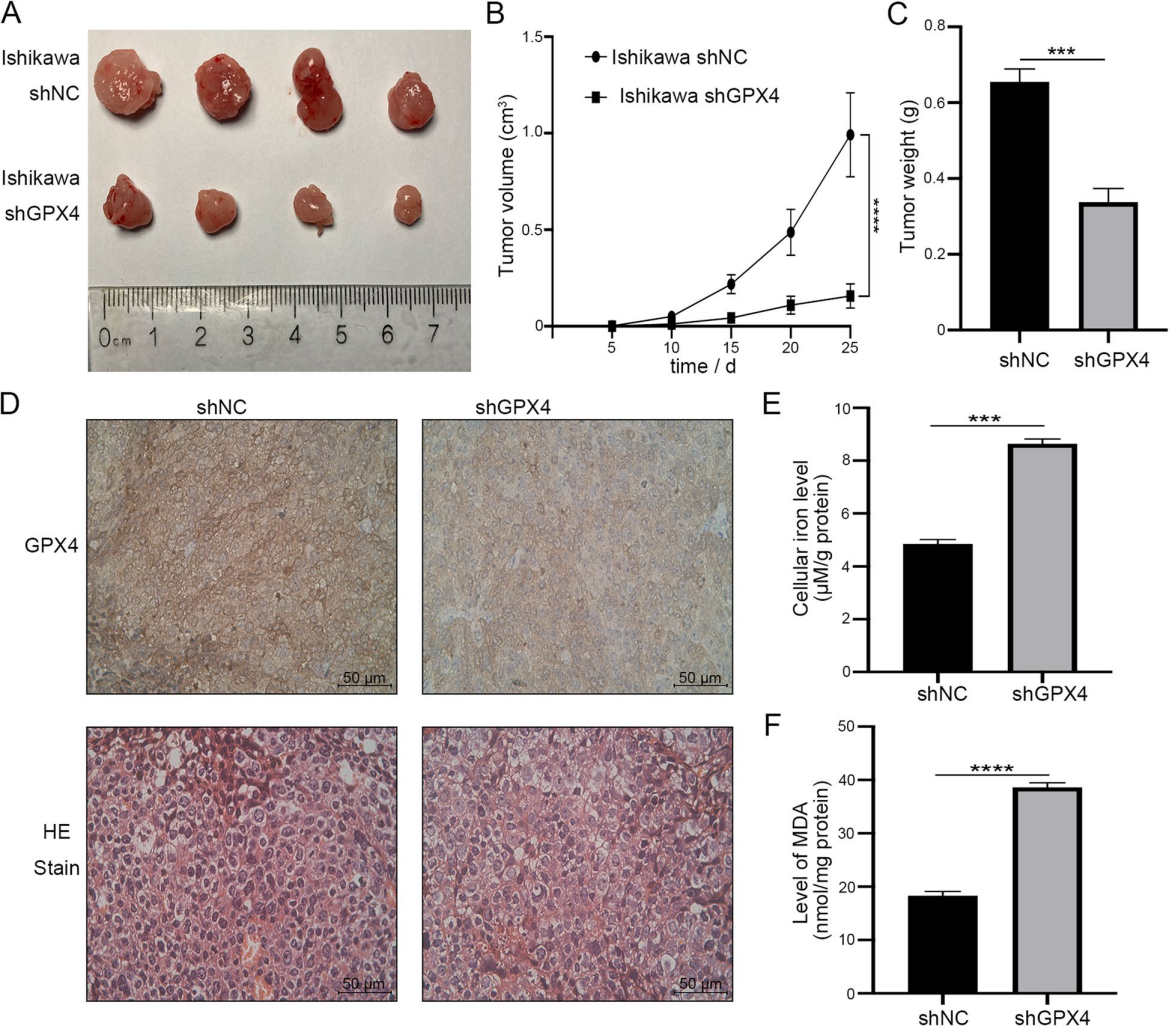
GPX4 promotes EC development, and GPX4 knockdown enhances ferroptosis activity in vivo. **A** Images of xenograft tumors from BALB/c-nude mice 25 days after the subcutaneous injection of stable GPX4 knockdown Ishikawa cells (shGPX4) or control cells (shNC). **B** Tumor volume was calculated according to the formula: (length × width^2^)/2. **C** Tumor weight of nude mice in shGPX4 and shNC group was assessed on day 25. **D** Immunohistochemistry and HE stain for GPX4 expression in xenograft tumors. Scale bar: 50 μm. **E** The concentrations of iron were evaluated in the tumor tissues. **F** The levels of MDA accumulation were evaluated in the tumor tissues

In order to verify the effect of GPX4 on endometrial cancer, an EC xenograft tumor model in nude mice was established to evaluate the effect of GPX4 knockdown in vivo. Ishikawa cells with stable GPX4 knockdown (shGPX4) or negative control (shNC) were injected subcutaneously into nude mice, respectively. The size of the tumor was measured every 5 days after cell injection, and the mice were sacrificed 25 days later. As shown in Fig. 5A, B, the inhibition of GPX4 drastically suppressed the tumor volume. The tumor weight of shGPX4 group also decreased remarkably after GPX4 knockdown (Fig. 5C). In addition, immunohistochemical results showed that the protein expression of GPX4 decreased significantly in shGPX4 group (Fig. 5D). Finally, we examined the levels of Fe^2+^ and MDA in tumor tissues. Compared with shNC group, after the knockdown of GPX4, the concentration of Fe^2+^ increased significantly (Fig. 5E) and the level of MDA increased significantly (Fig. 5F). All these data provide evidence that GPX4 could act as a tumor activator for the growth of EC, and that knockout of GPX4 enhances ferroptosis activity in vivo.

### ELK1 activates GPX4 transcription in EC cells

**Fig. 6:**
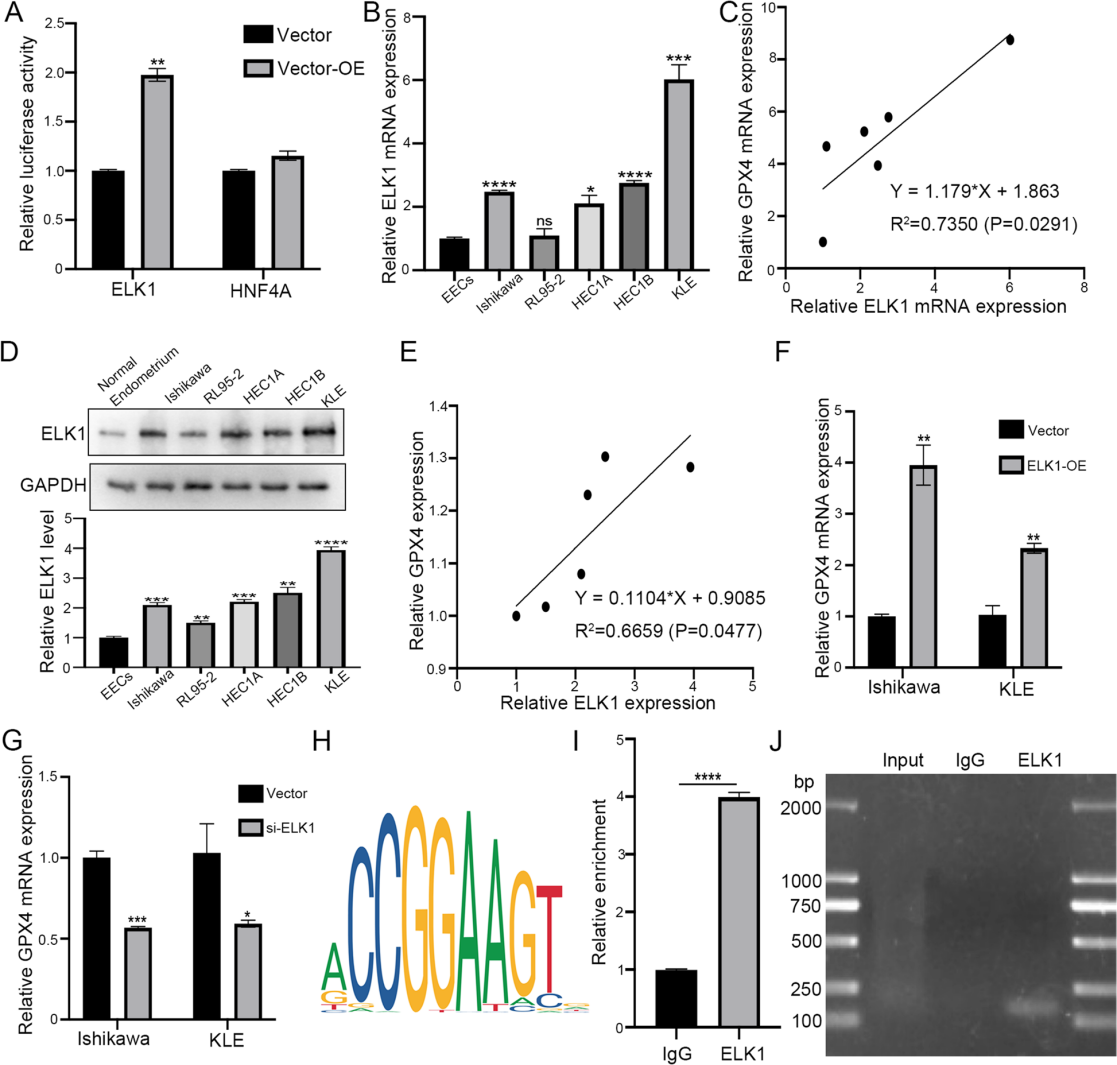
ELK1 binds directly to the GPX4 promoter to regulate the expression of GPX4 in EC cells. **A** Double luciferase reporter assay confirmed the interaction between ELK1 and GPX4 promoters in Ishikawa cells. **B** ELK1 mRNA expression in EECs and EC cell lines was analyzed by qRT-PCR. **C** Correlation between ELK1 and GPX4 in normal endometrial and EC cells in mRNA level was analyzed. **D** ELK1 protein expression in EECs and EC cell lines was analyzed by western blot. **E** Correlation between ELK1 and GPX4 in normal endometrial and EC cell lines in protein level was analyzed. **F** The expression level of GPX4 mRNA was identified by qRT-PCR after ELK1 overexpression. **G** The expression level of GPX4 mRNA was identified by qRT-PCR after ELK1 down expression. **H** The binding sites of ELK1 and GPX4 were predicted by the JASPAR database. **I** ChIP assay verified that ELK1 could bind to GPX4 promoter in Ishikawa cells. **J** The length of the qPCR product was tested by DNA gel

To further explore the upstream regulation of GPX4 in endometrial carcinoma, we used the JASPAR database (http://jaspar.genereg.net/) and PROMO software (http://alggen.lsi.upc.es/cgi-bin/promo_v3/promo/promoinit.cgi?dirDB=TF_8.3) to search for potential transcription factors in GPX4 promoter. Two candidates including ELK1 (ETS transcription factor ELK1) and HNF4A (hepatocyte nuclear factor 4 alpha) were obtained for further analysis. By using a luciferase reporter construct, the relative luciferase activity of GPX4 (within the 2000 bp promoter region) increased significantly only after transfection of ELK1 overexpression plasmid (Fig. 6A). We then analyzed the expression of ELK1 and GPX4 in EECs and five different endometrial carcinoma cell lines. qRT-PCR results showed a positive correlation between ELK1 and GPX4 in the mRNA level of EC cells (R^2^ = 0.8423, p < 0.05, Fig. 2A, Fig. 6B, C). The same results were observed in the protein level (R^2^ = 0.6659, *p* < 0.05, Fig. 2B, Fig. 6D, E). In addition, overexpressing ELK1 also significantly increased the GPX4 mRNA expression in Ishikawa and KLE cells (Fig. 6F). Correspondingly, the mRNA level of GPX4 decreased significantly after the knockdown of ELK1 (Fig. 6G). Therefore, ELK1 was identified as the most possible transcription factor that interacts with GPX4. Subsequently, the specific ELK1 binding sites on the GPX4 promoter were also predicted (Fig. 6H) and listed in Additional file 2: Table S1. According to the predicted binding sites, we designed ChIP-qPCR primers and performed the ChIP assay. The result indicated that ELK1 was significantly enriched in the GPX4 promoter in Ishikawa cells (Fig. 6I). DNA gel revealed that the length of the qPCR product was about 100—200 bp (Fig. 6J). Taken together, ELK1 could directly activate GPX4 transcription by binding to its promoter region in endometrial carcinoma.

### ELK1 / GPX4 axis is involved in the progression of EC by regulating ferroptosis

**Fig. 7:**
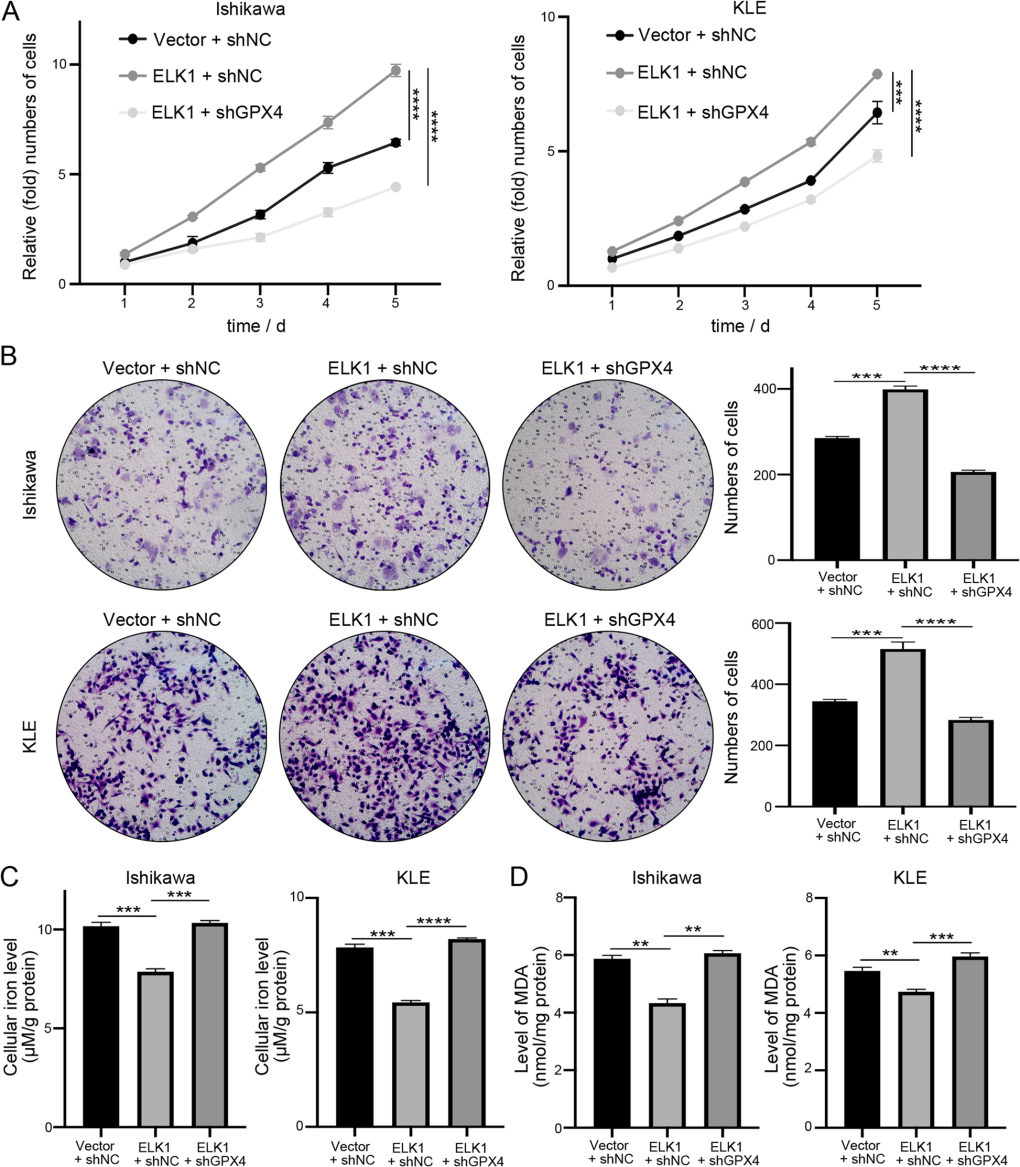
ELK1 / GPX4 axis is involved in the progression of EC by regulating ferroptosis. **A**,** B** Cell proliferation and migration ability were assessed by CCK8 and transwell assay after overexpression of ELK1 and downregulation of GPX4. **C**,** D** Ferroptosis activity was detected by the Fe^2+^ and MDA assay after overexpression of ELK1 and downregulation of GPX4

To demonstrate the effects of the ELK1 / GPX4 axis on endometrial carcinoma, ELK1 vector and GPX4 shRNA were co-transfected into Ishikawa and KLE cells. CCK8 and transwell assays confirmed that overexpression of ELK1 could promote cell proliferation and migration of EC cells (Fig. 7A, B). Rescue experiments identified that the enhanced cell proliferation and migration ability induced by ELK1 overexpression could be reversed by GPX4 knockdown (Fig. 7A, B). Next, we inspected the role of ELK1 in ferroptosis. After overexpression of ELK1, the intracellular Fe^2+^ concentration and MDA level were lower than those in the control group, but they could be reversed and increased after transfecting shGPX4 (Fig. 7C, D). The above data suggest that ELK1 could affect EC progression along with GPX4 by influencing ferroptosis activity.

## Discussion

The incidence rate and mortality of EC are increasing year by year, but there is still no effective treatment for advanced and recurrent EC. Recently, More and more studies suggest that the artificial introduction of ferroptosis is considered a promising treatment for cancers resistant to traditional therapies [27–29]. Ferroptosis is considered to be an iron-dependent oxidative cell death, which may be driven by the inactivation of GPX4. In addition, ferroptosis can be assessed by high intracellular Fe^2+^ levels and low GSH levels, accompanied by a series of oxidative stress [30]. Researchers have revealed that the ferroptosis process was aberrantly regulated in endometrial carcinoma and an activator of ferroptosis can induce cell death in EC cells [31, 32]. Despite that, the potential carcinogenic function and the exact mechanism of ferroptosis in EC still deserve further study.

In this study, we first demonstrated that the expression of ferroptosis “star regulator” GPX4 was up-regulated in EC after analysis of online databases and clinical samples. Inhibiting GPX4 expression could reduce the rate of EC cells proliferation and migration, increase apoptosis ratio, and arrest the cell cycle. Knockdown of GPX4 induced higher Fe^2+^, MDA, and ROS levels and decreased MMP, which activated ferroptosis. These data confirmed the basic role of GPX4 as a new target for EC tumor development and inhibition of Ferroptosis.

How to regulate GPX4 expression and activity has become a hot topic to explore the potential strategies for ferroptosis-related diseases nowadays. Supplementing intracellular selenium or glutathione can up-regulate GPX4 expression [33], while ferroptosis inducers such as ML162 [34] and RSL3 [35] can inhibit its activity. Post-translational modifications (PTMs), such as ubiquitination [36], succination [37], and alkylation [38] also can affect GPX4 protein level/activity. More and more studies have shown that transcription factors play an important role in the occurrence and development of cancers. Transcription factors can inhibit or enhance gene expression by interacting with CIS factors. Previous studies have shown that transcription factors can regulate the expression of ferroptosis-related proteins, such as transcription factor SP2 or Nrf2 transcriptionally regulate GPX4 protein level [39]. So, we wondered if there has potential transcriptional regulation that affects the GPX4 overexpression in EC.

We used the JASPAR database and PROMO software to predict the transcription factors that can regulate GPX4 expression. Two potential transcription factors including ELK1 and HNF4A were used as candidates. Through the double-luciferase reporter assay, only ELK1 showed high relative luciferase activity of the GPX4 promoter. The ChIP-qPCR analysis clearly showed that ELK1 can directly regulate its transcription by binding to GPX4 promoter. Nevertheless, the expression of GPX4 was up-regulated or down-regulated after overexpression or interference with ELK1, which further proved that the activity of GPX4 promoter induced by ELK1 was ELK1 dependent.

ETS Transcription Factor ELK1 (ELK1), as a transcription factor that triggers downstream targets including c-Fos proto-oncogene [40–42], is involved in regulating the expression of various genes associated with cell growth, proliferation, apoptosis, tissue remodeling, and angiogenesis. ELK1 enhances its binding activity to DNA through its ETS domain, so as to connect the target gene promoter to regulate the expression and activity of downstream proteins [43, 44]. A large number of studies have shown that ELK1 can be used as a cancer protein for many human cancers, including pancreatic cancer [45, 46], colorectal cancer [47, 48], gastric cancer [49], lung cancer [50], and nasopharyngeal carcinoma [51]. Considering the unique role of ELK1 in activating gene transcription related to cancer progression, we conducted an in-depth evaluation of its value in endometrial carcinoma and confirmed that ELK1 enhanced GPX4 transcription, inhibited ferroptosis, and promoted cancer progression.

In conclusion, our results suggest that GPX4 inhibits ferroptosis and promotes endometrial carcinoma progression through ELK1 transcriptional activation, and ELK1 / GPX4 axis is expected to become a new direction for the treatment of EC.

Finally, we found that there are still some limitations in our work. First of all, the EC samples we detected are relatively fewer and the pathological types only focus on endometrioid adenocarcinoma. Expanding the number of tissue samples and pathological types of EC should be better. Then, since we found and confirmed that ELK1 could transcribe and activate the expression of GPX4, it can be used as a new target to regulate ferroptosis. If a compound that can inhibit ELK1 is screened, it can promote ferroptosis to treat EC. However, due to technical limitations, we have not achieved this step. We hope that drugs or inhibitors targeting ELK1 will be invented in the future to help improve the survival of EC.

## Conclusion

In conclusion, the findings presented in this study provides a proof of concept for GPX4 as the key regulator of ferroptosis in EC and ELK1 as a positive regulator genetically upstream of GPX4. These findings implicate a ELK1 / GPX4 axis in the progression of EC and the role of ferroptosis resistance. Therefore, the mechanistic characterization of ELK1 / GPX4 axis may help to pave the way to develop a new therapeutic strategy for targeting ELK1 and its downstream effector GPX4 in patients with EC.

## Supplementary Information

**Additional file 1:** **Table S1**. The prediction binding domain between ELK1 and GPX4.

**Additional file 2.** Uncropped western blotting analysis.

## Data Availability

The raw data and materials supporting the conclusions of this article will be made available by the authors, without undue reservation.

## Abbreviations

GPX4: Glutathione peroxidase 4
EC: Endometrial cancer
IHC: Immunohistochemistry
qRT-PCR: Quantitative real-time polymerase chain reaction
GAPDH: Glyceraldehyde 3-phosphate dehydrogenase
ELK1: ETS transcription factor ELK1
ChIP: Chromatin immunoprecipitation
IARC: International agency for research on cancer
SI: Staining intensity
SDS: Sodium dodecyl sulfate
PVDF: Polyvinylidene difluoride
ECL: Enhanced chemiluminescence
CCK8: Cell counting kit-8
FCM: Flow cytometry
ROS: Reactive oxygen species
MDA: Malondialdehyde
MMP: Mitochondrial membrane potential
SEM: Standard error of the mean
HNF4A: Hepatocyte nuclear factor 4 alpha
