# Supplementary material for: GPX4 suppresses ferroptosis to promote malignant progression of endometrial carcinoma via transcriptional activation by ELK1
Source: BMC Cancer. 2022 Aug 12;22:881. doi: 10.1186/s12885-022-09986-3 (PMC9373394; doi:10.1186/s12885-022-09986-3)
Supplement: Supplementary file 1 — Additional file 1: Table S1. The prediction binding domain between ELK1 and GPX4. [file 12885_2022_9986_MOESM1_ESM.pdf]

Supplementary Table 2 The prediction binding domain between ELK1 and GPX4

Gene name	Start	End	Strand	Predicted sequence
ELK1	1845	1854	-	ACCGGACGTG
ELK1	1028	1037	+	GCCGGATGCG
