# Supplementary figures and images for: GPX4 suppresses ferroptosis to promote malignant progression of endometrial carcinoma via transcriptional activation by ELK1
Source: BMC Cancer. 2022 Aug 12;22:881. doi: 10.1186/s12885-022-09986-3 (PMC9373394; doi:10.1186/s12885-022-09986-3)

Fig. 1D

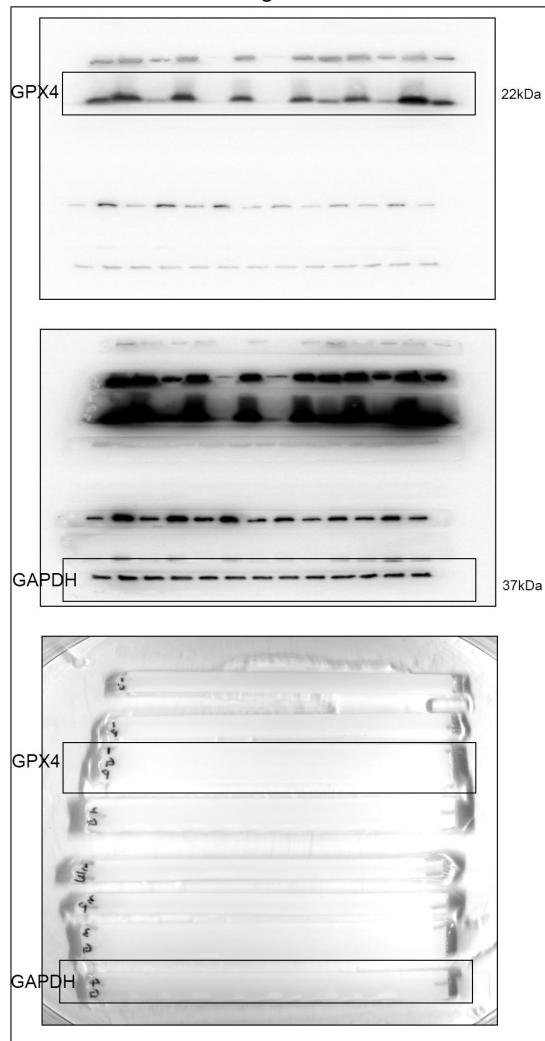

Fig. 2B

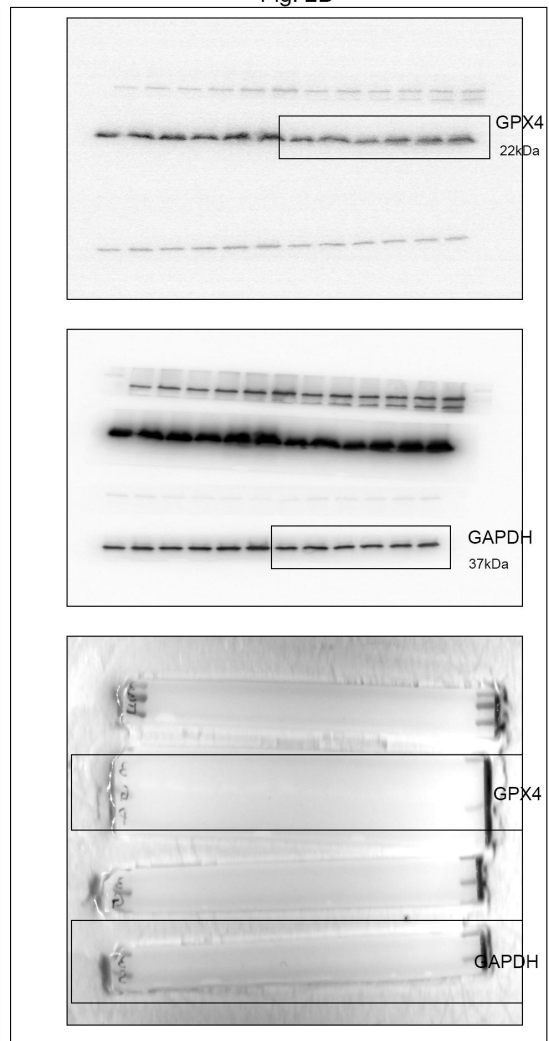

Fig. 2D

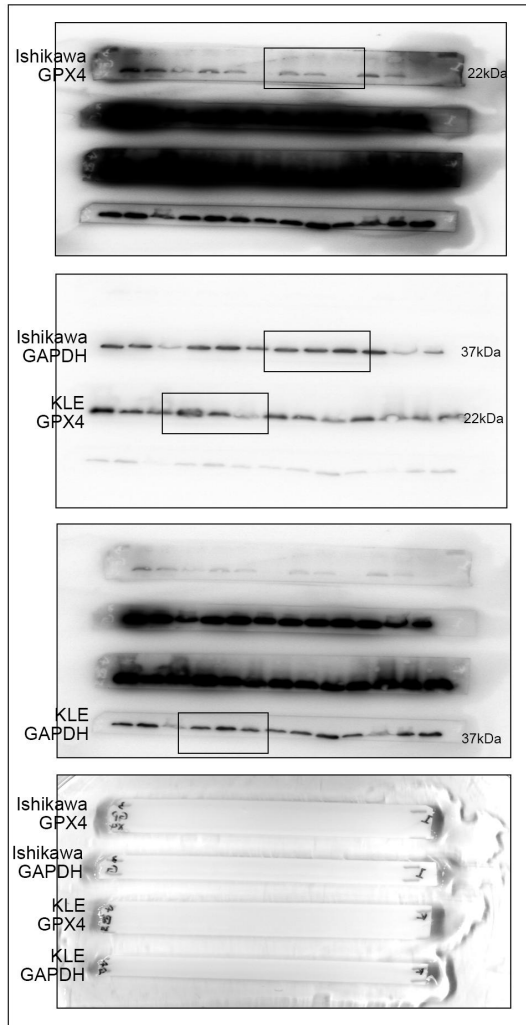

Fig. 6D

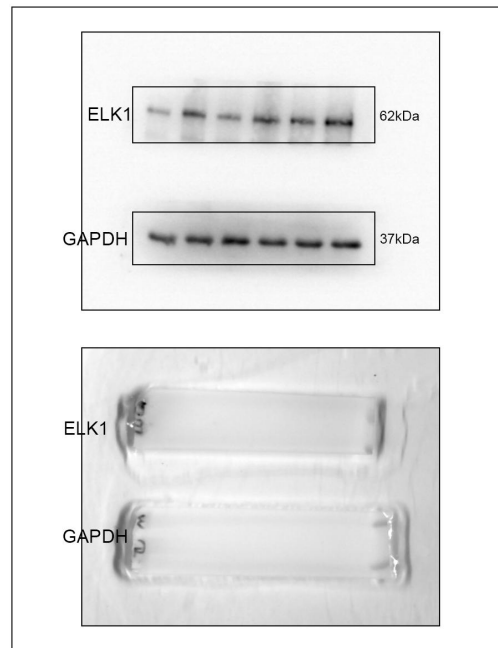

Fig. 6J

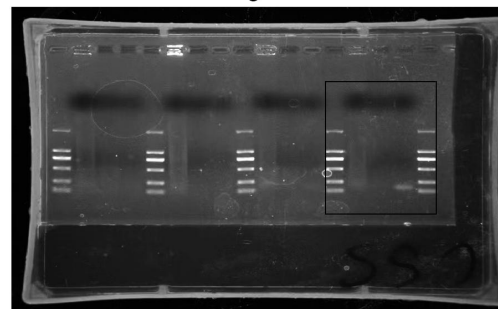

Supplement: Supplementary file 2 — Additional file 2. Uncropped western blotting analysis. [file 12885_2022_9986_MOESM2_ESM.pdf]
